# The Acute Toxicity and Hematological Characterization of the Effects of Tentacle-Only Extract from the Jellyfish *Cyanea capillata*

**DOI:** 10.3390/md9040526

**Published:** 2011-03-29

**Authors:** Liang Xiao, Sihua Liu, Qian He, Qianqian Wang, Xuting Ye, Guoyan Liu, Fei Nie, Jie Zhao, Liming Zhang

**Affiliations:** 1 Department of Chemical Defense Medicine, Faculty of Naval Medicine, Second Military Medical University, Shanghai 200433, China; E-Mails: xiaoliang830713@sohu.com (L.X.); langer1013@126.com (S.L.); abc_w@163.com (Q.W.); lgy_laurie@yahoo.com.cn (G.L.); niefei527@126.com (F.N.); 2 Department of Gynecology and Obstetrics, Shanghai Hospital, Second Military Medical University, Shanghai 200433, China; E-Mail: cpulj@126.com; 3 Department of Biophysics, School of Basic Medical, Second Military Medical University, Shanghai 200433, China; E-Mail: xuting_3@hotmail.com

**Keywords:** jellyfish, *Cyanea capillata*, tentacle-only extract, LD_50_, toxic symptom, hematological

## Abstract

To investigate the hematologic changes and the activities of jellyfish venoms other than hemolytic and cardiovascular toxicities, the acute toxicity of tentacle-only extract (TOE) from the jellyfish *Cyanea capillata* was observed in mice, and hematological indexes were examined in rats. The median lethal dose (LD_50_) of TOE was 4.25 mg/kg, and the acute toxicity involved both heart- and nervous system-related symptoms. Arterial blood gas indexes, including pH, PCO_2_, HCO_3_^−^, HCO_3_std, TCO_2_, BEecf and BE (B), decreased significantly. PO_2_ showed a slight increase, while SO_2_c (%) had no change at any time. Na^+^ and Ca^2+^ decreased, but K^+^ increased. Biochemical indexes, including LDH, CK, CK-MB, ALT, AST and sCr, significantly increased. Other biochemical indexes, including BUN and hemodiastase, remained normal. Lactic acid significantly increased, while glucose, Hct% and THbc showed slight temporary increases and then returned to normal. These results on the acute toxicity and hematological changes should improve our understanding of the *in vivo* pathophysiological effects of TOE from *C. capillata* and indicate that it may also have neurotoxicity, liver toxicity and muscular toxicity in addition to hemolytic and cardiovascular toxicities, but no kidney or pancreatic toxicity.

## 1. Introduction

Jellyfish may be the most common of the medically significant venomous marine creatures, especially in the tropic and subtropic regions. Jellyfish envenomation results in both local reactions (pain and red linear or hive-like lesions) and systemic reactions (nausea, vomiting, muscle cramps, diarrhea, dizziness, diaphoresis, coma, muscular spasms or even death) [1,2]. When human or animal victims contact jellyfish tentacles, the nematocyst threads will launch into their integument and inject venoms composed of multiple proteinaceous toxins into the victim’s body, leading to a variety of clinical symptoms, as mentioned above. Jellyfish venoms have a wide spectrum of biological activities, such as hemolytic, enzymatic, dermonecrotic, myotoxic, neurotoxic and cardiovascular toxic effects [2–4], among which the hemolytic and cardiovascular toxicities are the best studied, while the other activities are seldom mentioned. However, much about jellyfish venoms is still unknown.

We have previously found that the tentacle-only extract free of nematocysts (TOE) from the jellyfish *Cyanea capillata* showed similar hemolytic and cardiovascular toxicities to those of nematocyst venom [5–7]. Further studies have confirmed that cardiotoxicity is the main reason for death caused by TOE in rats [8], and hemolytic toxicity may act in synergy with the cardiovascular effects of TOE by inducing the release of K^+^ and lactic acid from the lysed erythrocytes [9]. Thus, in the present study, we adopted TOE from *C. capillata* as an abundant venom resource to survey the hematologic changes that occur in jellyfish envenomation and investigate the activities of jellyfish venom other than hemolytic and cardiovascular toxicities.

## 2. Results

### 2.1. LD_50_ Determination and Toxic Symptoms

**Figure 1.**
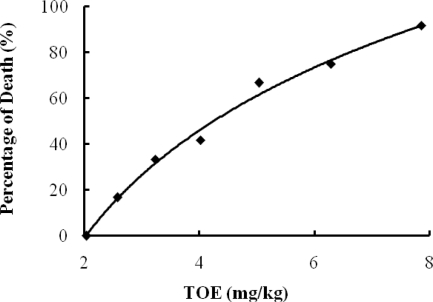
LD_50_ determination by tail vein injection in mice. The LD_50_ is 4.25 mg/kg for Kunming mice by tail vein injection (*n* = 12).

The LD_50_ of TOE was 4.25 mg toxin/kg body weight in Kunming mice by tail vein injection (Figure 1), and the toxic symptoms of the mice depended on the TOE dose (Table 1). At the lower doses (less than 5 mg/kg i.v.), the mice showed progressive malaise, slowness, crouching and weakness, but little change in breathing. Death mainly occurred within 48 h, and if the mice did not die within 48 h, they gradually returned to normal. Autopsy clearly showed heart enlargement and edema and lung edema and congestion with a bright red surface. At intermediate doses (5–10 mg/kg i.v.), deep and fast breathing was observed initially, and then breathing weakened gradually before finally arresting. Additionally, progressive malaise, slowness, crouching, muscle trembling and some double leg twitching were also observed, and most mice died within 2 h. At the higher doses (more than 10 mg/kg i.v.), deep and fast breathing occurred rapidly, and the mice also showed rapidly progressive whole-body trembling, double leg twitching, convulsion, opisthotonos and death. Most mice died within 5 min, and all of the mice died within 15 min. However, both the heart and lung were mostly normal on autopsy.

### 2.2. Effects of TOE on Arterial Blood Gas Indexes

TOE (5 mg/kg i.v.) produced significant effects on arterial blood gas indexes in anesthetized Sprague-Dawley (SD) rats. The pH and PCO_2_ dropped, while PO_2_ increased, and SO_2_c (%) was not obviously changed within 180 min after TOE injection. Other blood gas indexes, including HCO_3_^−^, HCO_3_std, TCO_2_, BEecf and BE (B), all decreased significantly within 5 min after TOE administration (Table 2).

### 2.3. Effects of TOE on Other Blood Indexes Determined by the Blood Gas Analyzer

The electrolytes Na^+^, K^+^ and Ca^2+^ were all significantly influenced by TOE (5 mg/kg i.v.). Na^+^ rapidly decreased and did not recover within 180 min. K^+^ rapidly increased, with a maximum at 5 min (11.7 ±1.9 mmol/L), then partially recovered. Ca^2+^ rapidly decreased and reached a minimum at 10 min (0.78 ±0.16 mmol/L), then increased to 1.26 ±0.05 mmol/L at 180 min. Similar results were obtained for the standard Ca^2+^, which was adjusted at pH 7.4 (Table 3).

After TOE administration (5 mg/kg i.v.), Glu increased and reached a maximum (11.4 ±3.2 mmol/L) within 20 min, then gradually dropped to normal. Lac significantly increased and reached a maximum at 10 min (5.9 ± 0.9 mmol/L), then partially recovered. Both Hct% and THbc increased and reached a maximum at 10 min (53 ±4% and 16.3 ±1.2 g/dL, respectively), then dropped to normal (Table 3).

### 2.4. Effects of TOE on Blood Biochemical Indexes

After TOE administration (5 mg/kg i.v.), the blood biochemical indexes, including LDH, CK, CK-MB, ALT, AST and sCr, significantly increased within 180 min, while BUN and AMY were not influenced at any time (Table 4).

## 3. Discussion

*C. capillata*, a relatively mild toxic jellyfish in comparison to *Chironex fleckeri* or *Physalia physalis*, is a common species of the coast of China [10]. Therefore, the clinical manifestations of *C. capillata* envenomation and physiopathologic research on this venom have a particular significance in China. In the present study, we aimed to explore the activities besides hemolytic [5] and cardiovascular [6,7] toxicities of *C. capillata* venom by observing the toxic symptoms and hematological changes caused by TOE.

At first, the acute toxicity of TOE was determined in mice by tail vein injection, and the LD_50_ was calculated as 4.25 mg/kg. During the LD_50_ determination, we observed that the toxic symptoms were dose-dependent, so we divided the doses into three grades according to the toxic symptoms (Table 1). In the lower dose grade (less than 5 mg/kg), the mice mainly showed heart failure-related symptoms, including congestion and edema in lung and edema in heart. In the highest dose grade (more than 10 mg/kg), the mice mainly showed typical central nervous system-related symptoms, including body trembling, double leg twitching, convulsion and opisthotonos. In the intermediate dose grade (5–10 mg/kg), the mice showed heart failure and nervous system-related symptoms simultaneously. In light of this, we speculate that the TOE may have strong and acute neurotoxicity in addition to its cardiovascular toxicity, leading to rapid death at high doses.

Among the arterial blood gas indexes, pH, PO_2_ and PCO_2_ showed irreversible changes beginning quickly after TOE administration at the dose of 5 mg/kg i.v., and other indexes, including HCO_3_^−^, HCO_3_std, TCO_2_, BEecf and BE (B), decreased significantly within 5 min, suggesting that metabolic acidosis developed quickly, which might be attributed to a direct and severe hemolytic reaction that led to the release of both lactic acid and K^+^. Eventually, PCO_2_ decreased because of compensatory hyperpnea, but PO_2_ and SO_2_c (%) did not decrease until rat death, suggesting that the respiratory response is not the major cause of death by TOE.

After TOE administration, Na^+^ and Ca^2+^ decreased rapidly, but K^+^ increased and reached a maximum at 5 min. These responses could be easily attributed to the severe hemolysis of TOE *in vivo*, which induces intracellular lactic acid and K^+^ release. However, the hemolysis of TOE *in vivo* may not be the only mechanism underlying electrolyte changes. Nematocyst venom from *P. physalis* markedly increases Ca^2+^ influx, Na^+^ influx and K^+^ efflux in a dose-dependent manner in cultured embryonic chick heart cells [11]. Another study [12] found that *P. physalis* nematocyst venom can induce Ca^2+^ influx into various kinds of cells, which are not limited to those from the cardiovascular system. *P. physalis* venom produces pore-like structures in intact cultured cells [13]. All of these results indicate that TOE may decrease circulating Ca^2+^ and Na^+^ and increase K^+^ by stimulating cellular Ca^2+^ and Na^+^ influx and K^+^ efflux through pores created in the cell membrane.

In this study, the elevation of all examined heart injury-related enzymes, including LDH, CK, CK-MB and AST, further indicated that direct cardiac toxicity is the leading cause of death and that a cardiac depressive response is the basic physiopathologic change caused by TOE in anesthetized rats [8]. Moreover, AST and ALT significantly increased, indicating that liver function might be influenced by TOE *in vivo*. Preliminary *in vitro* studies have shown that jellyfish nematocyst venoms are extremely toxic to hepatocytes by inhibiting their metabolic activities [14,15], which is consistent with our *in vivo* results with TOE from *C. capillata*. However, it should be noted that acute liver congestion, which could have resulted from acute total heart failure, may also have contributed to the elevation of AST and ALT. sCr markedly increased, but BUN remained normal. In addition, we have reported the negative results of kidney pathological examinations previously [8]. Thus, these results indicate that the kidney might function normally at least 3 h after TOE administration. The sCr elevation may suggest that TOE has muscular toxicity, but our data do not allow us to distinguish whether the sCr elevation was simply due to cardiac muscle injury or both skeletal muscle and cardiac muscle injuries.

Blood glucose had an initial transient rise and then lowered to the normal level gradually within 180 min. Nemocyst venom from the “Irukandji” jellyfish *Alatina nr mordens* can cause marked increases of plasma epinephrine and norepinephrine in anesthetized rats [16], so we presume that the fluctuation of glucose level could be attributed to catecholamines or components contributing to release of endogenous catecholamines. Similarly, the initial pressor responses caused by *C. fleckeri* and *C. barnesi* venoms are also attributed to the over-release of endogenous catecholamines in rats [17,18]. Mature erythrocytes of mammals have no mitochondria, so glycolysis is their only glucose utilization pathway. In this study, the quickly increased lactic acid might be explained as the consequence of hemolysis induced by TOE. On the other hand, lactic acid elevation can result in a pH decrease, metabolic acidosis and K^+^ elevation. After TOE administration, hemodiastase did not significantly increase, suggesting that pancreatic function remained normal. Hct% and THbc showed an initial increase, then a quick recovery, indicating that hypovolemic shock, characterized by loss of numerous extracellular fluids, was not the reason for hypotension caused by TOE *in vivo*.

## 4. Experimental Section

### 4.1. TOE Preparation from the Jellyfish *C. capillata*

Specimens of *C. capillata* were collected in July 2009 on the Sanmenwan coast of the East China Sea in Zhejiang Province, China. The removed tentacles were preserved in plastic bags on dry ice and immediately shipped to Shanghai, where the samples were frozen at −70 °C until use. The TOE devoid of nematocysts was prepared following the method described by us and other authors [7,19]. Briefly, frozen tentacles were thawed at 4 °C and immersed in filtered seawater at the mass:volume ratio of 1:1 to allow autolysis of the tissues for 4 days. The mixture was stirred for 30 min twice daily. The autolyzed mixture was centrifuged at 10,000× *g* for 15 min thrice. The resultant supernatant was the TOE.

All procedures were performed at 4 °C or in an ice bath. Before being used for injection, the TOE was centrifuged at 10,000× *g* for 15 min to remove the sediments, followed by dialysis against PBS (0.01 mol/L, pH 7.4) for over 8 h. The protein concentration in the preparations was determined with the Bradford method [20], with fetal bovine serum as the standard.

### 4.2. LD_50_ Determination and Toxic Symptom

Kunming mice (17–22 g, provided by the Laboratory Animal Center of the Second Military Medical University, Shanghai) were randomized into 10 groups (*n* = 12) for TOE administration by tail vein injection (the same injection volume at 0.1 mL per 10 g body weight, with different TOE concentrations). The dose ratio between the adjacent groups was 0.8. After TOE administration, the toxic symptoms were monitored for one week, and the numbers of both surviving and dead mice were recorded. The median lethal dose (LD_50_) was calculated using Karber’s method [21]. All animal experiments were approved by the Ethics Committee of the Second Military Medical University.

### 4.3. Hematological Indexes Determined by an Arterial Blood Gas Analyzer

Male SD rats (220 ±20 g, provided by the Laboratory Animal Center of the Second Military Medical University, Shanghai) were anesthetized with urethane (1.2 g/kg i.p.). Tracheotomy was performed to make the spontaneous ventilation easier. A heparinized catheter was inserted into the external jugular vein for intravenous administration of TOE (5 mg/kg). An arterial blood sample was drawn through a catheter that was inserted into the left femoral artery by a heparinized syringe. Afterwards, the syringe was sealed quickly with a rubber stopper. Hematological indexes, including blood gases (direct indexes: pH, PCO_2_ and PO_2_; indirect indexes: HCO_3_^−^, HCO_3_std, TCO_2_, BEecf, BE (B) and SO_2_c (%)), electrolytes (Na^+^, K^+^ and Ca^2+^), hematocrit (Hct), glucose (Glu) and lactic acid (Lac), were measured within 30 min by an arterial blood gas analyzer (GEM Premier 3000, International Laboratory, USA). Measurements were divided into six groups: 0 (pre-administration), 5, 10, 20, 60 and 180 min after administration of 5 mg/kg TOE.

### 4.4. Hematological Indexes Determined by an Automatic Biochemistry Analyzer

Arterial blood samples, which were acquired as described in Section 2.3, were centrifuged at 3000× *g* for 15 min, and the supernatant was collected as the serum, which was analyzed by an automatic biochemistry analyzer (AIRONE-200puls, Crony Company, Italy). Hematological indexes, including lactate dehydrogenase (LDH), creatine kinase (CK), MB isoenzyme of creatine kinase (CK-MB), alanine aminotransferase (ALT), aspartate aminotransferase (AST), serum creatinine (sCr), blood urea nitrogen (BUN) and hemodiastase (AMY), were measured. Measurements were divided into six groups: 0 (pre-administration), 5, 10, 20, 60 and 180 min after administration of 5 mg/kg TOE.

### 4.5. Data Analysis

One-way analysis of variance (ANOVA) was used. In all cases, statistical significance was indicated by *P* < 0.05. All quantitative data are expressed as means ±SD.

## 5. Conclusions

The present study on the toxic symptoms and hematological changes induced by TOE advances our understanding of the *in vivo* pathophysiological effects of jellyfish envenomation. In addition to hemolytic and cardiovascular toxicities, TOE from *C. capillata* may also exhibit neurotoxicity, liver toxicity and muscular toxicity, but no kidney or pancreatic toxicity.

## Figures and Tables

**Table 1.** Clinical manifestations after administration of tentacle-only extract (TOE) from the jellyfish *C. capillata* in mice.

**TOE**	**Symptoms**	**Time to death**	**Autopsy**	
			**Heart**	**Lung**
<5 mg/kg	Little change in breathing. Progressive malaise, slowness, crouching and weakness	Mainly within 48 h	Enlarged, edema	Bright red, edema and congestion
5 10 mg/kg	Early breathing deep and fast, gradually weakened, and finally arrested. Progressive malaise, slowness, crouching, muscle trembling and some double leg twitching	Mainly within 2 h	Enlarged, edema	Edema, some congestion
>10 mg/kg	Rapid emergence of breathing deep and fast. Progressive body trembling, double leg twitching, convulsion, opisthotonos and death	Mainly within 5 min, all within 15 min	Normal	Normal

**Table 2.** Effects of TOE from the jellyfish *C. capillata* on arterial blood gas indexes in anesthetized SD rats.

	**Direct indexes**			**Indirect indexes**					

	**pH**	**PCO_2_ (mmHg)**	**PO_2_ (mmHg)**	**HCO_3_^−^ (mmol/L)**	**HCO_3_std (mmol/L)**	**TCO_2_ (mmol/L)**	**BEecf (mmol/L)**	**BE(B) (mmol/L)**	**SO_2_c (%)**
0 min	7.37 ± 0.03	48 ± 4	78 ± 10	27.5 ± 3.1	25.9 ± 2.4	28.9 ± 3.2	2.1 ± 3.5	1.4 ± 3.0	95 ± 2
5 min	7.32 ± 0.07 **	41 ± 7 *	88 ± 12	20.8 ± 3.3 **	20.9 ± 2.7 **	22.0 ± 3.4 **	−5.3 ± 3.8 **	−5.1 ± 3.4 **	95 ± 2
10 min	7.25 ± 0.05 **	41 ± 4 *	93 ± 4 **	18.1 ± 3.2 **	17.9 ± 2.7 **	19.3 ± 3.3 **	−9.2 ± 3.8 **	−8.9 ± 3.5 **	96 ± 1
20 min	7.27 ± 0.06 **	41 ± 8 *	87 ± 8 **	18.6 ± 2.6 **	18.7 ± 2.0 **	19.9 ± 2.8 **	−8.3 ± 2.8 **	−7.9 ± 2.6 **	95 ± 2
60 min	7.27 ± 0.05 **	36 ± 3 *	89 ± 11 *	16.5 ± 2.4 **	17.3 ± 2.3 **	17.5 ± 2.5 **	−10.5 ± 3.1 **	−9.7 ± 2.9 **	95 ± 2
180 min	7.28 ± 0.02 **	34 ± 7 **	91 ± 11 *	16.6 ± 3.2 **	17.6 ± 1.9 **	17.7 ± 3.5 **	−10.1 ± 3.1 **	−9.3 ± 2.5 **	94 ± 4

Data are presented as means ±SD (*n* = 6,

* *P* < 0.05,

** *P* < 0.01 *vs.* 0 min).

**Table 3.** Effects of TOE from the jellyfish *C. capillata* on blood indexes as measured by an arterial blood gas analyzer in anesthetized SD rats.

	**Na^+^ (mmol/L)**	**K^+^ (mmol/L)**	**Ca^2+^ (mmol/L)**	**Ca^2+^ (7.4) (mmol/L)**	**Glu (mmol/L)**	**Lac (mmol/L)**	**Hct (%)**	**THbc (g/dL)**
0 min	143 ± 2	3.6 ± 0.2	1.18 ± 0.04	1.17 ± 0.05	8.1 ± 1.3	2.7 ± 0.7	47 ± 4	14.8 ± 1.1
5 min	129 ± 4 **	11.7 ± 1.9 **	0.82 ± 0.12 **	0.79 ± 0.12 **	9.3 ± 2.2	5.1 ± 0.7 **	51 ± 5	15.8 ± 1.5
10 min	133 ± 4 **	9.7 ± 2.0 **	0.78 ± 0.16 **	0.73 ± 0.15 **	10.0 ± 2.8	5.9 ± 0.1 **	53 ± 4 *	16.3 ± 1.2 *
20 min	134 ± 3 **	6.8 ± 1.1 **	1.07 ± 0.17	1.05 ± 0.14 *	11.0 ± 2.3 *	5.3 ± 0.7 **	52 ± 5	16.0 ± 1.4
60 min	135 ± 4 **	5.3 ± 1.5 **	1.25 ± 0.09	1.18 ± 0.10	10.9 ± 1.7 **	4.7 ± 0.7 **	49 ± 3	15.1 ± 0.7
180 min	133 ± 3 **	6.5 ± 0.8 **	1.26 ± 0.05 **	1.20 ± 0.05	9.5 ± 2.4	4.3 ± 1.0 **	46 ± 4	14.4 ± 1.3

Data are presented as means ± SD (*n* = 6,

* *P* < 0.05,

** *P* < 0.01 *vs.* 0 min).

**Table 4.** Effects of TOE from the jellyfish *C. capillata* on biochemical indexes in anesthetized SD rats.

	**LDH (U/L)**	**CK (U/L)**	**CK-MB (U/L)**	**ALT (U/L)**	**AST (U/L)**	**sCr (μmol/L)**	**BUN (mmol/L)**	**AMY (U/L)**
0 min	695 ±187	649 ±393	1067 ±227	46 ±8	114 ±55	22 ±4	5.1 ±1.0	1946 ±254
5 min	692 ±295	987 ±588	1211 ±303	59 ±13	157 ±33	25 ±11	6.3 ±1.3	2021 ±581
10 min	718 ±358	1113 ±539	1574 ±456 *	56 ±20	154 ±53	29 ±9	6.0 ±1.7	2099 ±494
20 min	760 ±496	1192 ±697	1826 ±616 *	68 ±19 *	185 ±80	28 ±4 *	6.7 ±1.5	2343 ±393
60 min	3171 ±4422	2085 ±2782	1889 ±607 *	166 ±235	362 ±372	36 ±9 **	5.1 ±1.6	2070 ±342
180 min	5816 ±5335 *	2231 ±1212 *	2772 ±885 **	354 ±257 *	785 ±201 **	57 ±19 **	6.8 ±1.8	1913 ±664

Data are presented as means ±SD (*n* = 6,

* *P* < 0.05,

** *P* < 0.01 *vs.* 0 min).
